# A critical review with emphasis on recent pieces of evidence of *Moringa oleifera* biosorption in water and wastewater treatment

**DOI:** 10.1007/s11356-022-19938-w

**Published:** 2022-05-18

**Authors:** Asmaa Benettayeb, Muhammad Usman, Coffee Calvin Tinashe, Traore Adam, Boumediene Haddou

**Affiliations:** 1 Laboratoire de Génie Chimique et de catalyse hétérogène, Département de Génie Chimique, Université de Sciences et de la Technologie-Mohamed Boudiaf, USTO-MB, BP 1505 EL-M’NAOUAR, Oran, Algeria; 2 Laboratoire Physico-Chimie des Matériaux – Catalyse et Environnement – LPCM-CE, Université des Sciences et de la Technologie d’Oran Mohamed Boudiaf (USTO-MB), BP 1505, El M’naouer, 31000 Oran, Algeria; 3 Institute for Water Resources and Water Supply, Hamburg University of Technology, Am Schwarzenberg-Campus 3, 20173 Hamburg, Germany

**Keywords:** Biosorption, *Moringa* species, *Moringa oleifera*, Heavy metal ions, Dyes, Mechanism

## Abstract

The increasing demand for using competent and inexpensive methods based on biomaterials, like adsorption and biosorption, has given rise to the low-priced alternative biosorbents. In the past few years, *Moringa oleifera* (MO) has emerged as a green and low-priced biosorbent for the treatment of contaminated waters with heavy metals and dyes, and given its availability, we can create another generation of effective biosorbents based on different parts of this plant. In this review paper, we have briefed on the application of MO as a miraculous biosorbent for water purification. Moreover, the primary and cutting-edge methods for the purification and modification of MO to improve its adsorption are discussed. It was found that MO has abundant availability in the regions where it is grown, and simple chemical treatments increase the effectiveness of this plant in the treatment of some toxic contaminants. The different parts of this miraculous plant’s “seeds, leaves, or even husks” in their natural form also possess appreciable sorption capacities, high efficiency for treating low metal concentrations, and rapid adsorption kinetics. Thus, the advantages and disadvantages of different parts of MO as biosorbent, the conditions favorable to this biosorption, also, the proposal of a logical mechanism, which can justify the high efficiency of this plant, are discussed in this review. Finally, several conclusions have been drawn from some important works and which are examined in this review, and future suggestions are proposed.

## Introduction

In the context of sustainable development and in parallel with the global economic crisis, the green chemistry “renewable substances” has caught the attention of various researchers around the world, particularly in developing communities. In general, the appearance of new water technologies known as “clean and friendly to the environment” using renewable and green materials of the biological entity to provide possibly the most efficient and convincing solution to the inherent problems in water and wastewater treatment.

In relation to this, over the last decade, many uncomplicated and captivating studies have been focused on the development of efficient bioprocesses relying on biomaterials including *Moringa* for the organic and inorganic metallic contaminant recovery/decontamination from the industrial effluent by biosorption (Pal et al. 2019; Benettayeb et al. 2017, 2021a, b, c; Hamza et al. 2020). Without forgetting that there are other types of effective treatment for the treatment of wastewater, such as photocatalyst (Islam et al. 2019).

The treatment cost of removing toxic heavy metals or dyes from real wastewater or even from synthetic aqueous solutions is very high. The management of this cost is related to the type of treatment applied to purify water but in the case of biosorption is substantially associated with the type of biosorbent. The treatment cost of the biosorption process in the elimination of pollutants can be managed efficaciously, especially when biosorbent is available at an attractive price.

So, biosorption is a physicochemical process wherein the uptake of a substance from a solution takes place by attachment to solid-phase biomaterial (Gadd 2009; Fomina and Gadd 2014). The biosorption has been effectively useful in the decontamination and the recovery of aqueous phase heavy metal ions and dyes (Awual 2015, 2016a; Hasan et al. 2021a). Several researchers have used effective materials to treat several toxic pollutants in wastewater, such as cerium(III) (Awual et al. 2013; Kubra et al. 2021), lead(III) (Awual 2016b; Benettayeb et al. 2017; Awual 2019a; Awual and Hasan 2019), copper(II) and phosphate (Awual, 2019b, c), cesium (Awual 2016c; Awual et al. 2020; Hasan et al. 2021b), selerium(IV) (Awual et al. 2015), and also some toxic dyes (Islam et al. 2021; Teo et al. 2022).

Knowing that the *Moringa* is a miraculous tree native to the foothills of the Himalayas (northern India, Pakistan, Bangladesh, and Nepal) and Africa (Anwar et al. 2007; Paliwal et al. 2011). The perspective sorption properties of all parts (seeds, leaves, barks, and husk) of this plant have been proposed by multiple research groups for the sequestration of the notable hazardous and toxic heavy metal ions (e.g., Cu(II), Ni(II), Pb(II), Cd(II), Hg(II)) in wastewater (Reddy et al. 2010a, b, 2012; Kebede et al. 2018; Çelekli et al. 2019; de Oliveira et al. 2019; de Bezerra et al. 2020; Gautam et al. 2020a). The metal ions have been declared as prioritized pollutants by many countries (Arora and Chauhan 2021). The increasing levels of toxic heavy metal ions generally discharged to the freshwater streams in developing countries, are continuously gaining tremendous attention due to their prominent adverse effects on receiving water bodies (Usman et al. 2018b). In order to provide the environmental sustainability of freshwater resources, environmental researchers are constantly interested in cheap and locally available biomaterials like *Moringa*, which are not only effective for water treatment but also resistant to use at different conditions of wastewater treatment. So far, researchers are trying to find new MO-based biosorbents to improve their pollutant removal/recovery capacity as well as their selectivity toward some toxic pollutants.

This review will enlighten the reader’s vision about the importance of the *Moringa* tree, citing its potential uses in various fields, especially their importance in the field of water treatment. So, this review regroups a succinct overview of important information related to the use of *Moringa* in modern applications, mainly the depollution using different MO-based adsorbents, their waste management capability, as well as search for optimal operational conditions related to its use in the former field of application. The regrouped information are summarized and holistically discussed in this review. Additionally, we propose a mechanism, which justifies its effectiveness with the precision of the groups/functions, which participate in these mixed biosorption mechanisms for some pollutants (CV, BG, and Pb(II)). In the end, the comments and recommendations on future developments are provided, and an attempt is made to identify gaps in knowledge and future perspectives on the use of MO derivatives.

### *Moringa* and its species

**Fig. 1:**
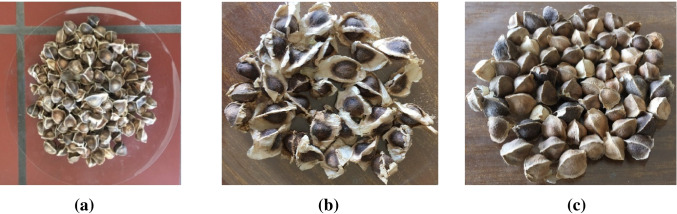
Photograph of the different unshelled seeds with husks available in Mali—from the second region of Mali in Dioila (Koulikoro): **a** *Moringa oleifera*, **b** *Moringa stenopetala*, **c** *Moringa drouhardii*

*Moringa* is the subject matter of this research review that is a source of proteins; mostly, its seeds are sources of proteins, lipids, fats, soluble vitamins, antioxidants, and other components (Saa et al. 2019). The different types of *Moringa* available in Mali such as *Moringa stenopetala*, MO, and *Moringa drouhardii* are shown in Fig. 1. Accordingly, Fig. 1 represents the different types of *Moringa* available in Mali.

**Fig. 2:**
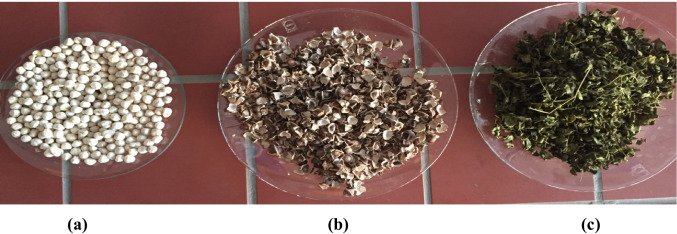
Photograph of the peeled MO seeds (MOS), MO husk (MOH), and MO leaves (MOL): **a** peeled MO seeds, **b** MO husk, **c** MO leaves

Different parts of the MO are represented in Fig. 2. All parts of the *Moringa* plant, particularly “MO leaves (MOL),” “MO seeds (MOS),” “MO bark *(*MOB),” and “MO husk (MOH)” are effective in the treatment of wastewater (Vieira et al. 2010; Soliman et al. 2019; Verma et al. 2020). Bearing in mind that this plant MO, especially its seeds MOS, as a good coagulant, is still being used till the current days (Taiwo et al. 2020; Vega Andrade et al. 2021). But usually, all the parts of this plant are used as natural biosorbents (Reddy et al. 2010a, 2012; Vieira et al. 2010; Acheampong et al. 2011; Mnisi and Ndibewu 2017; Adebayo et al. 2019; de Oliveira et al. 2019; Soliman et al. 2019; Verma et al. 2020).

### General uses of Moringa oleifera

As a mother’s best friend, MO is a tree with multiple benefits: medicinal, nutritional, and even industrial. Also, several authors have discussed its importance in food, animal feed, pharmacology, and cosmetics (Mahmood et al. 2010; Gopalakrishnan et al. 2016; 2019; Meireles et al. 2020). The main areas of this plant’s uses are (1) food; human nutrition and animal feed; (2) traditional medicines for the treatment of human diseases; (3) cosmetics and beauty products; (4) beekeeping; (5) soil protection; (6) water purification; (7) crop fertilization and biostimulant; (8) pesticides; (9) industry in various domain, and (10) landscape art.

**Fig. 3:**
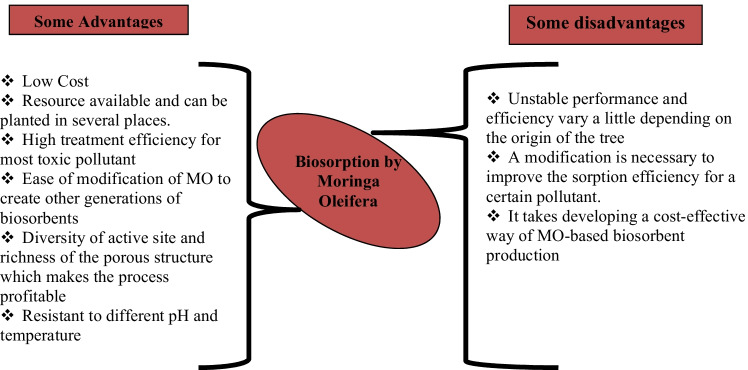
Main advantage and disadvantage of using the biosorption by *Moringa oleifera*

**Fig. 4:**
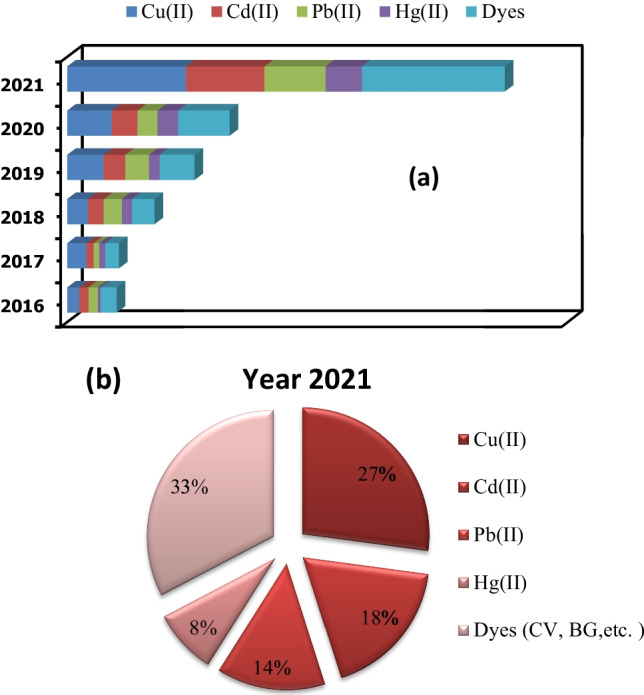
**a** A breakup of past studies from 2016 to 2021 on MO adsorption for the removal of heavy metals and organics dyes (updated on 03 December 2021) and **b** the number of MO adsorption studies published in 2021 on the removal of heavy metals and dyes

MO is a medicinal plant widely used in folkloric medicine of Africa and Asia for the treatment of ailments such as ulcer, wound, inflammation, heart problem, cancer, stroke, obesity, anemia, and liver damage (Aja et al. 2014). Also, traditional Indian medicine (Ayurvedic) says that MO can prevent 300 diseases and that its leaves have been exploited for preventive and curative purposes (Ganguly 2013). In addition, a study in India indicates that MO is one of the species used by traditional Siddha healers (Mutheeswaran et al. 2011). While the ancient Egyptians used MO oil on the skin due to its cosmetic properties (Mahmood et al. 2010). The details of the famous uses of MO are summarized in Table 1. The use of the natural grain of MO for adsorption of crystal violet (CV) and brilliant green (BG) is shown in Fig. 4, while the advantages and disadvantages of MO in biosorption are summarized in Fig. 3.

**Table 1:** Uses of MO in different areas

Areas	Advantage and benefits
*Food*	• The leaves are an exceptional superfood and an ideal nutritional supplement thanks to the high content of iron, protein, copper, various vitamins, and essential amino acids. • The leaves can be eaten fresh/powdered and can also be prepared in soup or in a salad (Broin 2012)
*Traditional medicine*	• Dried leaf helps in the fight against gastric ulcers, diarrhea, hypertension and hypotension, bronchopulmonary diseases, asthma, flu fever, etc. • The decocted flower is used against the flu.
*Medicine*	• All parts of MO have medicinal properties confirmed by experimental studies in different African, Asian, and Pan American countries (N. Kooltheat et al. 2014). • Used in the treatment of many diseases (Goyal et al. 2007) and also as an anti-neuralgic sinapism. • Used in Ayurvedic medicine and many other traditional medicines. • The richness of these leaves in flavonoids gives them a strong antimicrobial activity (Millogo-Kone et al. 2011)**.** • *M. oleifera* contains antioxidant compounds fighting against oxidative stress and vitamins: A, C, and E. • Root powder can help fight epilepsy, hysteria, hiccups, arthritis, kidney stones, rheumatism, fibroma, cysts, toothache, swelling of the feet and inflammation, disorders, and infections of the liver and spleen.
*Cosmetic*	• MOL powder is effective as hand hygiene soap. • Oil extracted from MOSs is a raw material in the cosmetics and perfume industry. • Oil is also used for hairstyles, the skin, and for the preparation of toilet soap and cosmetics.
*Water purification*	• The powder of MOS is a natural flocculant that can clarify cloudy water, thereby dissipating 99% of colloidal matter and 90 to 99% of bacteria. • The seeds contain a polyelectrolyte that allows the sedimentation of particles in suspension in water; these seeds constitute a first-rate coagulant for the treatment of river water having a high level of solid material in suspension.
*Therapeutic virtues*	• In Senegal, India, Benin, and Zimbabwe, the *Moringa* leaves are used in some programs to fight against malnutrition (De Saint Sauveur et Broin 2006) • The populations include the MOLs in the formulation of infant powder these leaves are considered a dietary supplement for infants (Madi et al. 2012). • In Senegal, Mansaly (2001*)* confirmed a marked improvement in the health of children with acute respiratory infections (ARI), measles, malaria, or diarrhea that are put on a MO diet (Mansaly 2001).
*Industry*	• The seeds contain 40% oil and 73% oleic acid; MO oil is used as a lubricant in fine machinery (William et al. 2015), like watchmaking (for its low tendency to deteriorate and become rancid and sticky). • It is also of interest in the cosmetics and perfumes industry (Foidl et al. 2001). • MO wood is an excellent pulp (Price 2007). • *Moringa* oil is therefore similar to higher quality oil such as olive oil. • Is used for the manufacture of rope, dye, and gum for tanning • *Moringa* oil can be used as edible vegetable oil and cooking oil (it goes rancid very slowly); as industrial oil; or as lighting oil in oil lamps because it produces a clear light that is almost smokeless, or as a base for fine paints.
*Pharmacological*	• Secondary metabolites are recognized by their numerous biological activities, which include antibacterial, anticancer, antifungal, analgesic, anti-inflammatory, gastrointestinal diuretics, and antioxidant activities (J. 1998; Bruneton 1999).
*Nutrition animal*	• Provides proteins, vitamins and minerals, and plant growth hormones to animals (livestock, fish) and also improves digestion of other foods • Promotes the health of farm and companion animals

### Some preparation methods of the Moringa oleifera leaf, seed powder, seeds pretreatment, and the protocol of extraction of the oils of the seeds

**Fig. 5:**
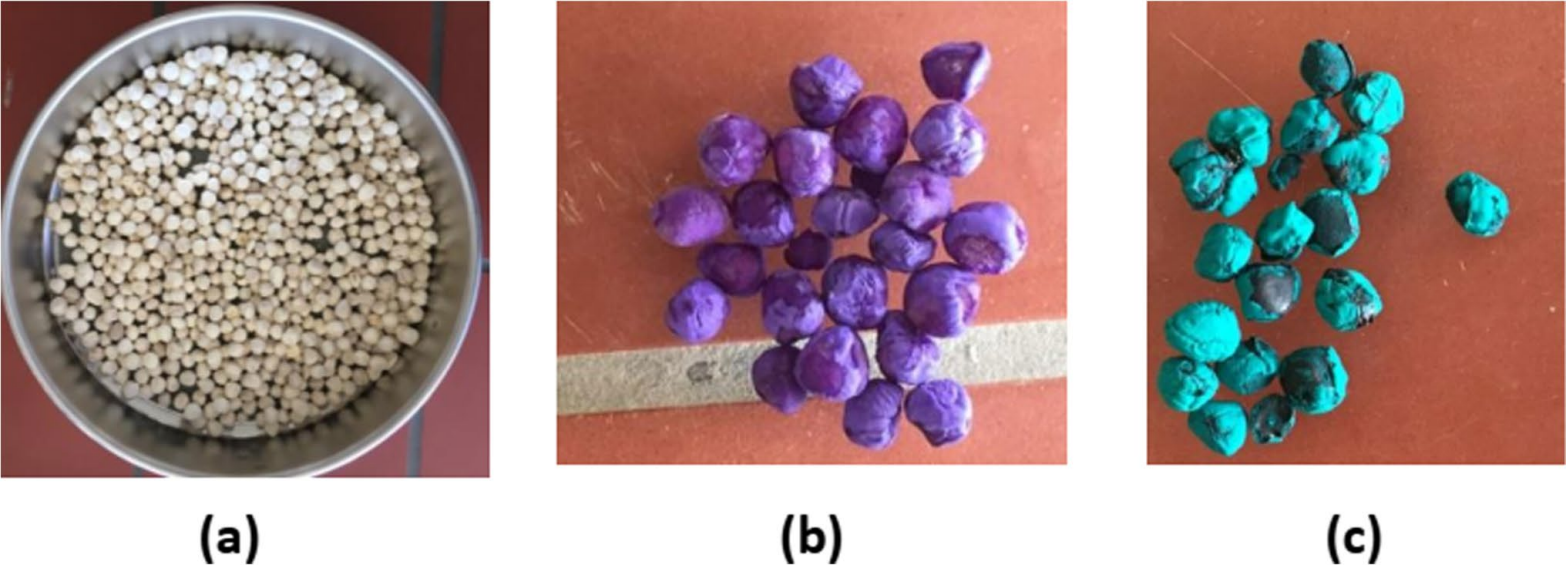
Complete seeds **a** before sorption and after 24 h of **b** crystal violet (CV) sorption and **c** brilliant green (BG)

The transformation process of MO is depending on its intended use. However, the main steps followed for the production of seed powder and husks, leave powders are dedicated in Fig. 5. For the production of the powder of leaves and husks, which was mentioned in the article by Benettayeb et al. (2021a, b, c), first, the seeds were shelled, and after, the leaves, seeds, and husks were washed with distilled water and dried in an oven at 40 °C for 48 h. Then grounded, and the powder obtained was dried at 50 °C for 30 min to reduce the residual moisture below 7.5% (Benettayeb and Haddou 2021).

The MO seeds contain a percentage of oil that can possibly be extracted. During oil extraction, the cake obtained as a by-product is very rich in protein and some of these proteins can be used as a source of flocculants and adsorptive materials for the sorption of various element toxic pollutants including heavy metals in water treatment (Baptista et al. 2017). Therefore, the method of oil extraction seems to be a decisive step for the intended use of the obtained cake as an adsorbent of pollutants.

For the preparation of MO seeds for oil extraction, the MO seeds are shelled classically by hand, and peeled seeds of the MO are washed carefully with distilled water to eliminate all traces of dirt. The washed peeled seeds “kernels” are dried in an oven at 40 °C for 24 h. The peeled MO seeds are pulverized with mortar and pestle, and the oil is extracted by Soxhlet extraction, and the separation of the oils/solvent is performed by simple distillation because the boiling temperature of the solvent used “hexane” is 69 °C (Benettayeb and Haddou 2021).

The resulting solid extracts are dried at room temperature in the open air for 1 h. The residue obtained after extracting the seed oil, generally known as “MO flour or MO powder,” is dried at 60 °C for 24 h and after is stored at room temperature. According to Benettayeb et al. (2021a, b, c), it has been found that the percentage of the protein content of the peeled MO seeds before and after oil extraction is different (Benettayeb and Haddou 2021). Indeed, increasing the rate of oil recovery allowed to increase the rate of protein in the residue, such an operation might favor the coagulant effect of MO flour solutions and increase the biosorption efficiency in most cases (Benettayeb and Haddou 2021).

#### *Moringa oleifera* for treatment of contaminants of water and wastewater

The use of MO as a biosorbent in ecological green processes to treat water, domestic wastewater, and industrial effluents has become increasingly popular as it is an environmentally friendly and innovative natural biomaterial. The biosorption process involving MO adsorption can offer many advantages such as cost reduction, reduction of by-product production as the cake generated during oil extraction will be utilized and increase the biodegradability over other decontamination processes (Villaseñor-Basulto et al. 2018) as well as the efficiency compared to other equivalent processes that utilize expensive organic products synthesized.

Aside from biosorption, which is the objective of this study, to eliminate the danger of toxic contaminants from water and wastewater, numerous treatment technologies have been used and developed in the decontamination/recovery of pollutants during the last few years. For example, ion exchange, biological treatment, coagulation (Mateus et al. 2018; Ngineering et al. 2019), membrane adsorption (Khulbe and Matsuura 2018), chemical precipitation (Son et al. 2020; Zhang and Duan 2020), solid-liquid separation using porous membranes (Usman et al. 2020a; Xu et al. 2021), membrane bioreactors (de Lopes et al. 2020), complexation (Trivunac and Stevanovic 2006; Deblonde et al. 2018), sorption on chemically synthesized and natural materials (Burakov et al. 2018; Ngabura et al. 2018; Šoštarić et al. 2018; Usman et al. 2018a), and reverse osmosis (de Lopes et al. 2020; Samaei et al. 2020; Cai et al. 2021) were used for wastewater treatment and decontamination. These technologies, except for adsorption, conventionally used in wastewater treatment, give low efficiency, complex installation, high operating costs, high energy requirements, and the generation of toxic secondary sludge (Araújo et al. 2010b, 2018). However, due to the new periodic demands and requirements, adsorption is the most widely used one because it still offers several advantages (Naushad 2014; Albadarin et al. 2017) (see Fig. 3) and is still known as a potential technology that is generally accepted as environmentally friendly and inexpensive for water treatment due to its ease of operation and maintenance, cost-effectiveness, simplicity of design, high efficiency and performance potential, flexibility, speed, and availability of diversified sorbents effective for each pollutant (Gopalakannan and Viswanathan 2015).

Despite being the simplest operation for treating toxic pollutants, the effectiveness of this technology strongly depends on the environment of the solution (temperature of the water, which intervenes in the matrix of the adsorbent, pH) and adsorptive medium (biomaterial and the products involved in the synthesis of the adsorbent) applied (Peng and Guo 2020). The efficiency of the adsorptive materials like MO is too sensitive to some parameters including nature and the origin of biosorption material, particle size, effective surface area, surface charge density, and pretreatment before use*.*

### Moringa **o**leifera as miraculous biosorbent

The data used to create Fig. 4a and b was collected from Scopus using the keywords of *Moringa* adsorption or removal of pollutants by *Moringa* and *Moringa* as potential adsorbents for metals and dyes. In the period 2016 to 2021, the Scopus database yielded 819 papers that investigated the potential of *Moringa* as an adsorbent for the removal of heavy metals and organic dyes. Figure 4 demonstrates that the use of *Moringa* as an adsorbent is increasing, especially since the number of studies published in 2021 is 347 (compared to the year 2016 with 60 papers). Figure 4b exhibits that it is mostly applied for decontamination of dyes (BG, CV) followed by Cu(II) ions and after Cd(II) ions, these data show that this plant is very important in the fields of adsorption, and can be used to create other generation of bioadsorbents (Benettayeb and Haddou 2021).

MO biomass has also been reported to bind some pollutants such as cationic dyes and metal ions through amino and carboxylic groups present in proteins, sulfonic groups (–SO_3_^−^), and other constituents in the seeds or the leaves of this plant. Figure 5 presents an example of MO seeds biosorption comparison before and after 24 h of biosorption of crystal violet (CV) and brilliant green (BG).

**Fig. 6:**
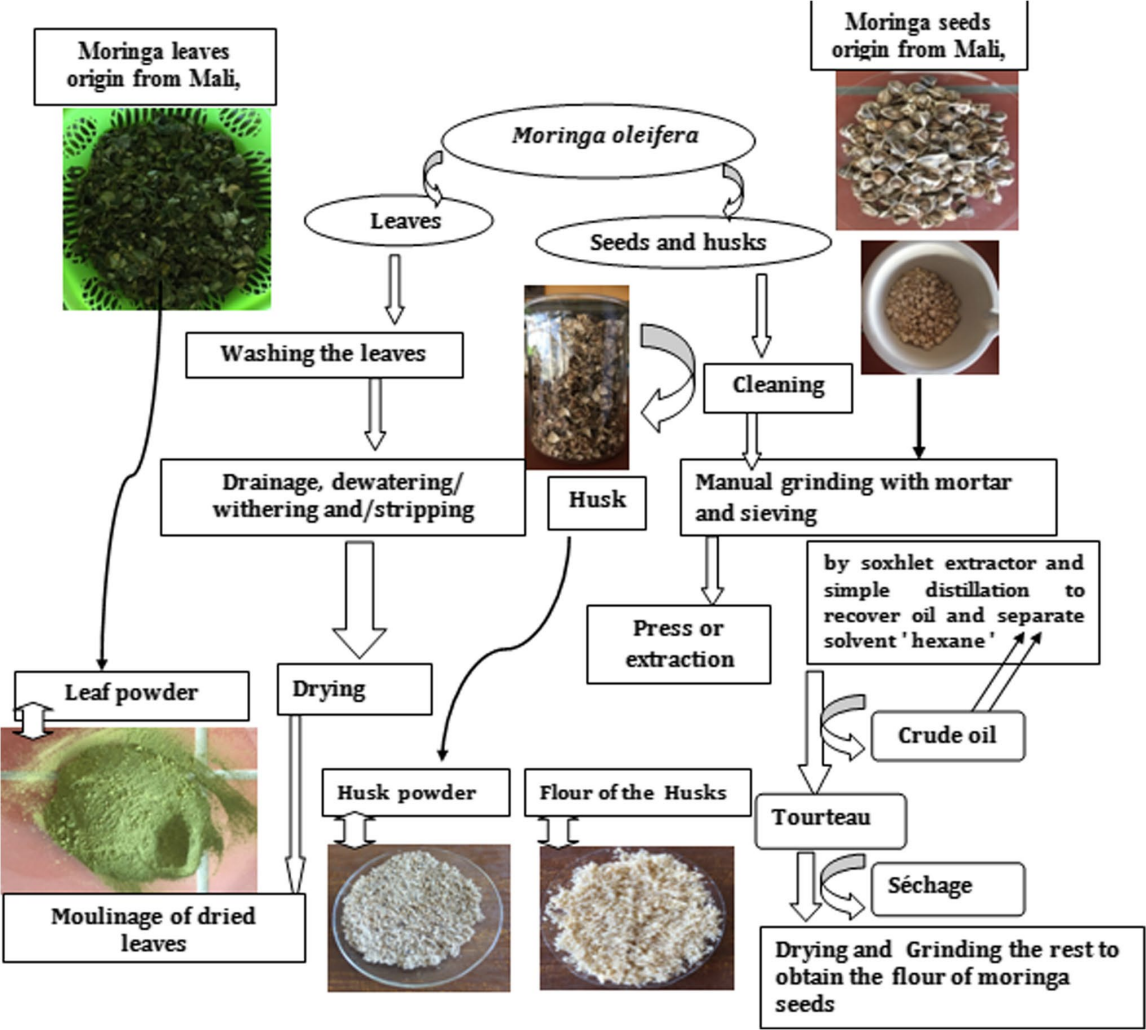
Diagram summarizing the process of production MO powder from MOS, MOL, and MOH

The functional groups on the surfaces of the biosorbents, especially function –N, –S can easily adsorb heavy metal ions, these functions provide selective and effective adsorption for various metal ions, which belong to class B such as (Hg(II), Ag(I), Pd(II), Pt(IV), Pt(III), Au(III), Cs(I)), and intermediate (borderline) according to the classifications of Pearson (1990) and (Nieboer and Richardson 1980). Also, MO contains a high percentage of lignin that is a heterogeneous complex biopolymer molecule, which is endowed with many different functional groups, such as methoxy, hydroxyl-aliphatic, carboxyl, and phenolic (Eun and Rowell 2005). For example, during the introduction of the −NH_2_ groups into the structure of the alginate by simple voices, an improvement in sorption for the ions of Pb(II), Cd(II), and Cu(II) was noticed (Benettayeb et al. 2017). Lin et al. demonstrated that the adsorbents with amine groups have special properties that enable them to adsorb compounds with cationic or anionic charges at different pH values (Lin et al. 2011). Similar conclusions were drawn by Sahebjamee et al. (2019). These studies confirm the importance of –NH_2_ in the adsorption of metals ions and toxic dyes on adsorptive materials including MO. Further studies revealed that the MO biomass residue can be used for the removal of pharmaceutical molecules such as diclofenac (DCF) from water (Araujo et al. 2018), which gave a *Q*_max_ of 72.8 mg g^−1^ obtained by electrostatic attraction between the negative groups (O^−^, OH^−^, and Cl^−^) of the DFC and the functional positive groups present on the surface of the adsorbent at a pH value of 5 (Araujo et al. 2018). The virgin MO seeds and husk as biosorbents have been exploited for the adsorption of trihalomethanes (Okoya et al. 2020). Table 2 summarizes the adsorption capacities of MO including all parts of it and its other species for the removal of some undesired pollutants from wastewater. Figure 6 diagram summarizes the process of production of the MO powder (MOP) from MOS, MOL, and MOH represented in Benettayeb and Haddou (2021).

**Table 2:** Biosorption ability of MO toward various toxic pollutants

Material	*Q*_max_ (mg/g)	Model	Mechanisms of adsorption	Source
CAMOL	209.5	Langmuir	The results indicate that the adsorption of Pb(II) onto CAMOL might be attributed to the chemical ion-exchange mechanism.	Reddy et al. (2010a)
CAMOL	171.4 167.9 163.9	Langmuir	Biosorption using the bonding of metal ions to carboxyl groups. According to the results of authors, Cd(II), Cu(II), and Ni(II) adsorbed mainly on active groups such as hydroxyl groups (–OH) and carboxyl groups (–COO)	Reddy et al. (2012)
PODA PODB	16.2 38.5	Langmuir	As for PODA, low energy is involved in the process, thereby leading to biosorption process characteristic of physisorption, but for the PODB is the opposite implies high energy in the interaction process biosorbent-adsorbate is the chemisorption.	de Oliveira et al. (2019)
MOS	70.5	Langmuir	The process comprises physical and chemical adsorption. The authors indicated that the major process is ion exchange, and the electrostatic attraction and ionic diffusion play an important role in the adsorption process.	Swelam et al. (2018)
MOB	34.6	Langmuir	Coordination of the Pb^2+^ ions with hydroxyl, carboxyl, and carbonyl groups present on the surface of MOB.	Reddy et al. (2010b)
MOB	30.4	Langmuir	The kinetic studies revealed that the biosorption process followed the pseudo-second-order kinetic model.	Reddy et al. (2011)
MOS	29.6	Langmuir	No indication of the mechanism in this work	Marques et al. (2012)
MOS	412.3	Langmuir	Physisorption nature	Çelekli et al. (2019)
MOH	14.7	Langmuir/sips	Physisorption nature (ΔH° < 40 kJ/mol)	de Bezerra et al. (2020)
MOS	23.1	-	No indication of the mechanism in this work but confirm the surface heterogeneity	Araújo et al. (2010a)
MOF	5.6	Langmuir	No indication of the mechanism in this work	Gautam et al. (2020a)
MOB	25.2	Langmuir	No indication of the mechanism in this work	Mnisi and Ndibewu (2017)
MOP and MOH	9.6	Langmuir	No indication of the mechanism in this work	Adebayo et al. (2019)
MOS	238	Langmuir	SEM-EDX analysis confirmed an exchange of Mg(II) and K(I) for Cu(II) on MOS and the binding energy for the ion exchange mechanism is 8 to 9 kJ mol^−1^	Acheampong et al. (2011)
SMOS	90.3 % for 25 mg/L of methylene blue	-	At a pH value of 6.5, the carboxylic groups are deprotonated and are negatively charged. These negatively charged carboxylate ligands are likely to attract the cationic dye species.	Raj et al. (2013)
SMOS	98.5 % for 25 mg/L of congo red	-	At lower pH of 2.5, the sorbent is positively charged due to the protonation of amino groups of the amino acids leading to the electrostatic attraction between the biomaterial and anionic dye.	Raj et al. (2013)

### Moringa oleifera as a coagulant

Also, MO is known for its attractive properties in wastewater treatment by coagulation, flocculation, and biosorption; this concept will reduce the environmental impact of hazardous chemicals used for the purification of contaminated water. Therefore, it might be a remedial solution in rural areas facing water scarcity where there are no resources to obtain expensive conventional techniques and materials. Mataka et al. reported that *Moringa stenopetala* flour showed better coagulant performance compared to MO in the removal of Pb(II) at pH 10.0, a dose of 15 g/L and 100 °C (Mataka et al. 2006). However, Obuseng et al. have obtained 95% removal efficiency of Pb(II) in water having a pH value of 2.0, a dose of coagulant 5 g/L, and a temperature of 22 °C using MO seed flour (Obuseng et al. 2012). Another research performed by Kebede et al. (2018) studied the elimination of Cd(II), Pb(II), and Cu(II) ions from industrial effluent using *Moringa stenopetala* seed powder in its natural form. The maximum Langmuir adsorption capacity (*Q*_max_) was found to be 233, 16.1, and 10.2 mg g^−1^ for Cd(II), Pb(II), and Cu(II), respectively, and reported adsorption efficiencies of all pollutants were in the range of 99–100% from synthetic wastewater. The removal efficiencies in complex wastewaters comparing industrial effluent were approximately 92–95% (Kebede et al. 2018).

#### Effect of modification of *Moringa oleifera* on some famous toxic pollutants

The structure of the biosorbent plays an important role in the biosorption process. The smaller is the pore size, the larger is the contact area of the biosorbent. It is therefore realistic to use a predominantly microporous biosorbent to obtain a good adsorption capacity as is the case with MO. The manufacture of bioadsorbents based on the different parts of MO varied from simplicity (simple modification method) to the synthesis of biosorbents using the complex method (several cascade reactions, to mix the properties of several materials).

The modification of MO-based biosorbents has been made to improve their affinity toward target pollutants and uptake rate as well as to improve their selectivity and to optimize (for example, surface area, adsorption capacity) or expand (for example, the composition of surface functional groups to remove the metal ions that are less or not adsorbed by pristine MO). There are several ways, which can be undertaken for the modification of MO. Indeed, modification methods include physical treatment modification, chemical treatment modification, functionalization/grafting/cross-linking, and impregnation. After this modification, the obtained results depend on the modification agent and the modification way. Table 2 shows the adsorption capacities of both pristine and modified MO-based biosorbents.

Tables 2 and 3 demonstrate that the nature and intrinsic characteristics of MO leaves, seeds, and husks have an effect on biosorption; habitually, the researchers apply pretreatment processes to modify or affect some of their properties inherent in biomaterials in order to promote the biosorption process. According to these researchers, the pretreatment processes that have been frequently practiced by various research groups include grinding, boiling, autoclaving, and acid/alkali processing (Reddy et al. 2010a, b, 2011, 2012; Araújo et al. 2010a; Acheampong et al. 2011; Marques et al. 2012; Raj et al. 2013; Mnisi and Ndibewu 2017; Adebayo et al. 2019; Çelekli et al. 2019; de Oliveira et al. 2019; Gautam et al. 2020a).

**Table 3:** Biosorption ability of *Moringa* toward various toxic pollutants

Material	Modification	Pollutants	pH	Temp. (°C)	Dose (g/L)	Equilibrium time (min)
CAMOL	MOL was treated with 0.1 N of NAOH, and after this, biomass-named BWMOL was mixed with citric acid (CA) to obtain CAMOL	Pb(II)	5.0	40 °C	-	120 min and 200 rpm
CAMOL	Chemical modification of *M. oleifera* leaves (MOL) using NaOH followed by citric acid treatment	Cd(II) Cu(II) Ni(II)	5.0	40 °C	0.04 g/L	120 min and 200 rpm
MPODA MPODB	MPODA; *Moringa* powder obtained through acidic chemical functionalization MPODB; *Moringa* powder obtained through basic chemical functionalization	Pb(II)	5.5	25/Δ*H*° value reveals the exothermic nature of the process	-	30 min and 200 rpm
MOS	*Moringa oleifera* seeds (MOS) without modification	P(II)	7.0	277 to 313 K endothermic process	0.7 g/L	5–300 min and 100 rpm
MOB	Biomass of *Moringa oleifera* bark (MOB) Chemical modification with double distilled water	Pb(II)	5.0	25 °C	0.4 g/L	30 min and 300 rpm
MOB	Chemical modification with double distilled water For removal of water-soluble color compounds	Ni(II)	6.0	50 °C	0.1–0.8 g/L	180 min and 300 rpm
MOS	Unshelled seeds were ground to obtain the size of 0.5 and 1.0 and were treated with 0.1 mol/L NaOH	Ni(II)	4.0–6.0		40 g/L	5 min
MOS	*Moringa oleifera* seed without modification particle size <125 μm	RR 120	1.0	50 °C	0.5 g/L	1440 min and 150 rpm
MOH	Chemical treatment with 0.1 M methylic alcohol and 0.1 M nitric acid and a thermal treatment was performed after these steps	Diuron	5.0	45°C	0.02 g/L	900 min
MOS	MOS without modification particle sizes from 75 to 500 μm	Ag(I)	6.50	-	100 g/L	20 min
MOF	Fruits of *M. oleifera* without modification sizes from 75 μm	Pb(II	5.8–6.0	27 ± 4 °C	-	120 rpm
MOB	MO bark without modification	V(V)	5.0	20 °C	0.150 g/L	100 rpm
MOP and MOH	Activated MO pod (MOP) and husks were carbonized at 450 °C for 30 min and were later activated with 0.1 M phosphoric acid.	Methylene blue (MB)	6.0	27 °C		30 min
MOS	Without modification, the MOS were washed and dried particle size of 0.5–0.8 mm	Cu(II)	7.0	30 ± 0.2 °C	2 g/L	1440 min and 100 rpm
SMOS	Shelled *Moringa oleifera* seed powder (SMOS) particle size (105 μm)	Methylene blue (MB) Congo red (CR)	6.5 2.5	Room temperature	4 g/L	40 min

Various researchers have cited the contributions of the chemical modification even though it was a simple chemical treatment and their positive effect on the structure of MO. In the work of de Bezerra et al. (2020) for the removal of diuron, a herbicide from contaminated water, the MO husk (MOH) powder, was chemically treated with two acids. After this chemical treatment, a thermal treatment was performed as a second step to remove some inorganic or organic matters from the MO surface. These matters are considered impurities, which interfere in the interaction of the contaminant with the surface of the biosorbent and increase the surface area (de Bezerra et al. 2020). Cusioli et al. explored the chemical modification of MO lam seed husks. The chemical modification (by 0.1 M of CH_3_OH for 4 h in a 1:5 m/v ratio and 0.1 M of HNO_3_ for 1 h) following thermal treatment of produced biomaterial was carried out for 12 h. The chemical modified MO powder was subsequently used for atrazine removal. It was proven to be effective by demonstrating *Q*_max_ of 10.321 mg/g under the following conditions: temperature of 318 K, pH of 5, and a mass of 0.04 g. The equilibrium was reached at 1200 min, and the authors confirmed that the process of biosorption occurs by chemisorption and physisorption mechanism together (Cusioli et al. 2019).

According to the work of Bhatti et al*.,* the efficient removal of zinc from aqueous solution was observed using MO lam. The MO lam was chemically pretreated, and the obtained sorption capacity was about 52.1 mg/g (with an efficiency of 90% at a time of 50 min). The adsorption test was investigated at 30 °C and pH of 7.0 (Bhatti et al. 2007). In another study, MOL were used as an effective biosorbent for treating solutions having low concentrations of Cd(II) (1, 3, 5 up to 7 mg L^−1^) without a modification or any kind of chemical treatment, the authors confirming that this powder has a considerable high biosorption capacity (Ali et al. 2015).

In the work of Gautam et al., the authors made new magnetic nanoparticles based on MO, which have proven to be effective for the biosorption of lead ions. The results obeyed the Freundlich isothermal adsorption with a maximum capacity of 64.97 mg/g (with an efficiency of 94.08%) at a temperature of 50 °C, pH 5.0, and a time of 60 min (Gautam et al. 2020b). To improve the chelation of heavy metal ions such as Pb(II), Cd(II), Cu(II), and Ni(II) from aqueous in batch systems studies, it has been found that citric acid and NaOH modification of MO enhances the uptake of these metal ions (Reddy et al. 2010a, 2012).

The researchers tested the efficacy of MO seeds (natural, particle sizes from 75 to 500 μm) toward Ag(I), Cd(II), Co(II), Cu(II), and Pb(II) at a concentration of 5.0 mg L^−1^, 2 g of adsorbent, contact time of 20 min, a temperature of 25 °C, and pH of 6.5. The best efficiency was achieved for Ag(I), and the efficiency varies from 28 for Co(II) to 100% of Ag(I) and demonstrate following the order: Ag(I) > Pb(II) > Cu(II) > Cd(II) > Co(II) (Araújo et al. 2010a). In this work, the seeds were dried at 65 °C for 24 h and used directly without any further treatment (Araújo et al. 2010a).

The biosorption of Pb(II) by MOL has been as well and obtained *Q*_max_ is 45.8 mg/g (at an adsorbent dosage 1.5 g/L), while this biosorbent achieved 98.6% removal of Pb(II) at an MOL dose of 10 g L^−1^ and pH 6. After five biosorption/desorption cycles (using 0.3 M HCl solution as desorbing agent), an 8% decrease in the removal efficiency of MOL for Pb(II) was realized (97% to 89%) (Imran et al. 2019).

Several ways of modification have been used in the manufacture of new materials based on polysaccharides “such as alginate, chitosan,” but in the case of MO, despite the richness of this plant (source of different protein), little work has been done to find new ways to modify this plant. A comparison was made by Abdeen (2018) between the biosorption efficiency of chitosan and MO against some metal ions and stated that the affinity of MO seeds was in the following order: Cd(II) > Mn(II) > Cu(II) > Ni(II) > Zn(II) > Fe(II) > Pb(II). While the affinity of chitosan was Cd(II) > Cu(II) > Ni(II) > Zn(II) > Mn(II) > Fe(II) > Pb(II). The elementary analysis of *Moringa* seeds and chitosan showed, respectively, a percentage of –N of 6.2 and 7%, but for the elements Ca, Mg, and Na, the chitosan is rich compared to *Moringa* (Abdeen 2018). The highest percentage removal of Fe, Zn, Mn, and Cu by using MOS extract was 45.74% Fe, 46.15% Zn, 64.29% Mn(II), and 47.37% Cu(II). Other studies demonstrated the same outcomes (Sajidu et al. 2005; Subramanium et al. 2011). There is a new method of making MO beads from Algerian olive oil using a cold gelation method and MO from Mali; these beads were based on MOS, MOH and MOL, and gelatin. It was shown that beads own great efficiency with respect to CV, BG, and Pb (II) ions at the range of a pH value of 5–6. The removal efficiencies varied from 38 to 83% depending on the modification and the pollutant type. It is relevant to state that the EDX analysis showed the highest percentage of amino groups and the FT-IR spectra showed the existence of various functional groups such as, –NH, –OH, and –COOH (Benettayeb and Haddou 2021).

## Methods for efficient characterization of biosorption

### Analytical characteristics

The characterization of different MO products is essential for an attempt to explain their behavior, thus establishing a complete data sheet for each solid requires a series of analyses such as X-ray diffraction (XRD), Fourier transformed infrared spectroscopy (FTIR), scanning electron microscopy-energy dispersive X-ray spectroscopy (SEM-EDX) analysis, and ATD/ATG thermal analysis.

This list of analyses depends on the type of biomaterial considered, its origin, the type of modification carried out as well as the envisaged application. For example, in the case of MO leaves modification to obtain a nanopowder, researchers carried out some specific analyses like SEM/SEM-EDX, Brunauer, Emmett, and Teller (BET) surface area, FTIR, and XRD.

Several analytical methods have been used to characterize this type of biosorbent: FTIR analysis, the morphology, and semiquantitative composition of the materials synthesized have been visualized by SEM. In several works, the composition of the beads was determined using SEM-EDX.

Generally, analytical methods like; FTIR, SEM/TEM, and SEM-EDX are needed to understand the morphology, composition, and distribution of elements in the adsorbent surface. The importance of FITR spectra has increased in the sorption domain and particularly in the biosorption because they can facilitate understanding the sorption mechanism and steps involved, as the identification of functional groups (active sites) in biosorbent suggests the ability to make bonds with them in the biosorption process, so we can understand approximately how each pollutant makes the choice of active sites/adsorbents.

Knowing that, FTIR spectroscopy provides a means of measuring low-frequency vibrational movements, which are useful for characterizing many organometallic and inorganic molecules. The results of these measurements often allow one to approximate the chemical nature and molecular structure of an organometallic complex. Also, the FTIR analyses before and after biosorption are very important because the change in transmittance in FTIR spectra for metal-loaded MO indicates that these functional groups are involved in biosorption. (Ahmady-Asbchin et al. 2008) confirmed that biosorption of metals to biosolids results in a decrease in absorbance compared to the raw sample.

The analysis TGA/DCS supplies information about the thermal stability of biosorbent (Fomina and Gadd 2014). According to Araújo et al. (2010a), the thermogravimetric analysis was used to characterize the decomposition stages and thermal stability of the MO seeds, some methods adopted by these researchers for the characterization of MO seeds are FTIR spectroscopy, thermogravimetric analysis (TGA), XRD, and SEM and confirmed that these are sufficient for this type of biosorbent.

#### Physical characterization

Physical characteristics such as humidity rate, swelling rate, amino rate, real density, apparent density, total pore volume, and chemical porosity for the precision of the functional groups in the surface of prepared materials are determined by using a simple protocol. Generally, the titration methods can give important information about the existence of some active sites (or some components like –NH_2_, –COOH, and phenolic content) in the surface of the biosorbent and their concentration/quantity.

- Estimation of humidity rate and swelling/deswelling rate

In order to determine the percentage of water content (W%) maintained intrinsically in the structural form (for example in the beads), while determining the percentage of swelling (S%), the beads are left for 24–72 h in distilled water. The sample is weighed when it is wet, then after 72 h reweighed, and the moisture content (W%) is calculated by Eq. 1. For the % swelling rate, the sample is weighed when it is dried, then after 24–72 h weighed again, and the rate of swelling is calculated by Eq. 2.

1 $$W\%=\frac{100\times \left({W}_{\mathrm{w}}-{W}_{\mathrm{d}}\right)}{W_{\mathrm{w}}}$$

2 $$S\%=\frac{\left({W}_{\mathrm{w}}-{W}_{\mathrm{d}}\right)}{W_{\mathrm{w}}}\times 100\kern0.5em$$

where *W*_W_: mass or weight of wet beads after 72 h and *W*_d_: mass or weight of the beads after 72 h of drying.

b) Estimation of real density, apparent density, total pore volume, and chemical porosity

While for the actual density, a small amount of known mass (g) is put into a tared pycnometer, which is filled with methanol. Knowing the volume of the pycnometer${\boldsymbol{v}}_{\mathbf{m}}=\frac{{\boldsymbol{m}}_{\mathbf{2}}}{{\boldsymbol{\rho}}_{\boldsymbol{m}}}$, the mass of the material used and the density of methanol (*ρ*_m_), makes it possible to calculate the real density (*ρ*_réelle_) of the biomass using Eqs. 3 and 4.

3 $${V}_{\mathrm{R}}={V}_{\mathrm{t}}-{V}_{\mathrm{m}}\kern2.5em$$

4 $${\rho}_{\mathrm{real}}=\frac{m_1}{V_{\mathrm{R}}}\kern5em$$

where *m*_l_ and *m*_2_ represent the mass (g) of material and methanol, respectively; *m*_t_ represents total mass (material + methanol); *V*_t_ represents the total volume of the pycnometer, *ρ*_m_ denotes the density of methanol at measurement *T* and *P* (25 °C and 1 atm) = 0.7864 g/cm^3^; *V*_m_ represents the volume of methanol used; and *V*_R_ represents the actual volume of the material.

For apparent density, the method used consists of introducing a quantity of sample corresponding to any apparent volume into a previously washed and dried test tube. To prevent the powder from sticking to the walls of the specimen, it is necessary to shake the assembly in order to obtain a good settlement of the latter. Knowing the mass of the sample (*m*_1_) and the apparent volume (*V*_app_) provides access to the apparent density (*ρ*_*app*_) of the material. The total pore volume of the sample “V.P.T” is calculated from the apparent and real densities (see Eq. 6).

5 $$\rho \mathrm{app}=\frac{m_{\mathrm{l}}}{V_{\mathrm{app}}}$$

6 $$\mathrm{V}.\mathrm{P}.\mathrm{T}=\frac{1}{\uprho_{\mathrm{app}}}-\frac{1}{\uprho_{\mathrm{real}}}$$

For the determination of active sites and the calculation of the percentage of functional groups like –NH_2_, –COOH, and other groups on the surface of MO before and after modification can be performed the analytical methods like volumetric titration.

c) Estimation of content of carboxylic groups

Various analytical methods have been used by several researchers in order to know the percentage of new functions or those existing in the adsorbent. In the case of the estimation, by calculation, of the content of carboxyl function, the methods reported in (Atia et al. 2005c, a) can be exercised. For instance, (Atia et al. 2005c, a) reported that 0.005 g of the biosorbent is stirred in 50 mL of 0.2 M aqueous NaOH solution at a temperature of 25 °C for 6 h. The mixture is then filtered, and the concentration of unreacted NaOH is determined by titration of 20 mL of the filtrate with 0.2 M HCl. Therefore, the concentration of carboxyl groups (mmol/g) in the solid is calculated by Eq. 7, knowing that, the *M*_1_ and *M*_2_ are the initial and final concentrations of NaOH, *V* is the volume of NaOH, and *W* is the weight of the adsorbent.

7 $$\mathrm{Content}\ \mathrm{of}\ \mathrm{carboxyl}\ \mathrm{groups}\ \left(\mathrm{mmol}/\mathrm{g}\right)=\left(M1-M2\right)\times \frac{V}{W}$$

d) Estimation of the content of amine groups

The amine content of chemical materials including biosorbents has been estimated using a volumetric method (Atia et al. 2005b; Donia et al. 2012; Elwakeel et al. 2014). Briefly, 50 ml of 0.2 M HCl solution is added to 0.005 g of biosorbent for 15 h providing constant stirring. The residual HCl concentration is estimated by titration against the 0.2 M NaOH solution using phenolphthalein as a colored indicator. The number of moles of HCl having interacted with the amine groups, and consequently, the concentration of amine groups (mmol.g^−1^) is calculated from Eqs. 8 and 9.

8 $$\mathrm{Concentration}\ \mathrm{of}\ \mathrm{amine}\ \mathrm{groups}\ \left(\mathrm{mmolof}\ \mathrm{solution}/\mathrm{g}\ \mathrm{of}\hbox{--} \mathrm{N}\right)=\left(\mathrm{M}1-\mathrm{M}2\right)\times \frac{50}{0.005}$$

9 $$\mathrm{Amine}\ \mathrm{group}\ \left(\%\right)=\mathrm{concentration}\ \mathrm{in}\ \mathrm{amine}\ \mathrm{group}\mathrm{s}\ \left(\mathrm{mmol}/\mathrm{g}\ \mathrm{of}\ \mathrm{N}\right)\times \mathrm{conversion}\ \mathrm{coefficient}$$

e) Estimation of the total content of phenolic

Gautam et al. (2020b) adopted a procedure for the determination of the total phenolic content of MO by preparing extracts of its leaves. In the first step, 10 g of dried fine leaf powder was mixed with 200 ml of ethanol in a volumetric flask for 24 h at room temperature. The resulting solution was filtered using filter paper and dried the supernatant in a vacuum at 40 °C using a rotary evaporator. The second step is the mixture of 0.5 mL of synthesized plant extract in 2.5 ml of 10% Folin–Ciocalteu’s reagent and 2.5 mL of 7.5% NaHCO_3_ and kept the same for incubation at 45 °C for 1 h. Blank was also prepared in a similar way.

The absorbance (*λ*_max_) of the sample was determined at 760 nm. The sample concentration was determined from the profile of the gallic acid calibration curve using a gallic acid concentration of 0.01 to 0.12 mg/L, and the total phenolic content was measured using Eq. 10 (Gautam et al. 2020b).

10 $$\mathrm{Total}\ \mathrm{phenolic}\ \mathrm{content}\ \left(x\mathrm{p}\right)=\frac{C\times V}{M}$$

where *C* is the concentration of the calibration curve derived from the standard curve equation: *Y* = *ax* + *b*; *Y* is the absorbance at 760 nm; and *x*_p_ is the total phenolic content, *V* (mL) is the volume of extract used, and *M* (g) is the mass of the extract used. The total phenolic content was calculated to be 111.61 mg/g of gallic acid equivalent. Knowing that, we must obtain a calibration curve with an *R*^2^ greater than 0.999.

## Effect of operational parameters in biosorption by *Moringa oleifera*

The biosorption process can be carried out in two modes: batch mode and continuous mode (column). The batch process is frequently used to perform biosorption processes on a laboratory scale. However, most industrial applications prefer continuous mode biosorption. However, batch experiments are used to assess the basic information required such as the efficiency of the biosorbents toward a specific substance, optimal experimental conditions (contact time, Temperature, initial adsorbate concentration, amount of biosorbent applied, and other parameters), the biosorption rate and the possibility of spent biosorbent regeneration.

**Fig. 7:**
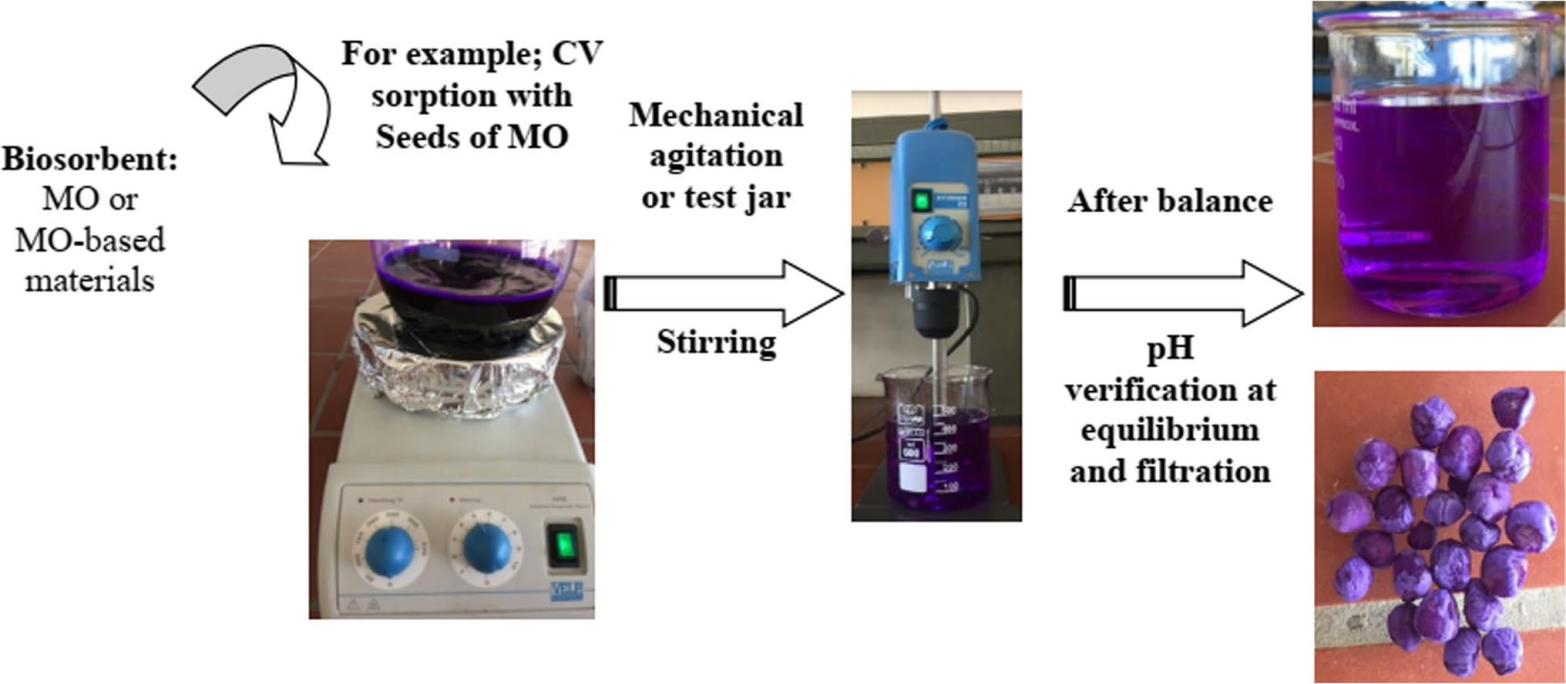
Descriptive diagram of the batch adsorption process by MO seeds, for example, for the adsorption of the dye, crystal violet (CV)

Also, the size of the molecule and ion acting as adsorbate can greatly influence the adsorption process by their arrangement on the surface of the material because the particles with low molecular mass are light and move faster. The so-called batch method is shown schematically in Fig. 7. When the peeled MO seeds in their natural form are applied we can use magnetic agitation or even use mechanical agitation.

### Effect of pH

It is a prerequisite to know the efficiency of the biosorption of biosorbents at different pH conditions to determine the effective range of pH on the biosorption in order to carry out this step.

Choosing the right pH value for each material can affect the biosorption process because the variation in pH conditions of water can cause group modification as well as affect the surface charge of biosorbents and consequently, increase or decrease the sorption efficiency. Therefore, the value of pH plays a major and direct role in the study of pollutant sorption phenomena. The value of pH can affect the solution chemistry of the pollutant, the activity of the functional groups in the biomass, and the competition of ions for binding sites (Adeniyi and Ighalo 2019).

In general, the surface charge potential of the adsorbents, the protonation/deprotonation behavior of the adsorbate, and the interaction mechanism between the undesired adsorbate and the adsorbent used to change depending on the pH of the water being processed for decontamination. These parameters are the decisive factors influencing the biosorption process. It should be noted that the solution pH is an important criterion since it may affect the metal/dyes speciation through the formation of complexes (in presence of ligands), which, in turn, influence the binding mechanism. Furthermore, it can also induce precipitation phenomena (Benettayeb et al. 2017). Besides, the solubility of the metal ions will depend on the total metal concentration and the equilibrium pH value. Indeed, sorbent-solution interaction may increase the pH of the solution (by proton binding, for example) (Benettayeb et al. 2017).

Cr(VI) adsorption was studied as a function of pH over a range of 2–8 for *Moringa stenopetala* seed powder (MSSP) at an initial concentration of 30 mg/L wastewater. The results of the experiment indicate that optimal Cr(VI) removal efficiency of MSSP was obtained at pH 2.0 for 120-min contact time. It is evident that Cr(VI) removal efficiency increases with a decrease in pH. The favorable effect at low pH might be due to the neutralization of negative changes on the surface of *Moringa* by excess hydrogen ions, thereby facilitating the diffusion of the hydrogen chromate ion (HCrO_4_^−^) (Badessa et al. 2020).

Most researchers use NaOH to raise the solution pH. However, other bases are preferred because the addition of NaOH can result in the formation of insoluble complexes, which causes precipitation at low pH values and low concentrations of undesirable substances. Thereby, deformation of the sorption results occurred.

### pH of zero point charge (pH_pzc_)

The initial pH value of the solution is an important factor affecting the sorption of the pollutant. This parameter can change the adsorbent surface density. The pH_pzc_ (known as pH at point of a zero charge) corresponds to the pH value for which the charge on the material surface is zero. Classically, the pH_PZC_ is reckoned using the “pH drift method” (K. Kadirvelu and C. Faur-Brasquet 2000). This method states that the pH_pzc_ is the point where the initial pH curve as a function of the final pH intercepts the line corresponding to pH_initial_ = pH_final_; in fact, the pH_pzc_ represents the border where the surface charge is zero and changes sign. The pH_zpc_ also can be determined by following the variation of pH_i_ = f(pH_eq_) during the isotherm studies, pH_pzc_ corresponding to the point of intersection of the experimental curve (final pH as a function of initial pH) with the linear plot (pH_i_ = pH_f_). This method has been used in many studies (Lopez-Ramon et al. 1999; Kadirvelu and C. Faur-Brasquet 2000; F. Boudrahem and F. Aissani-Benissad 2011).

**Fig. 8:**
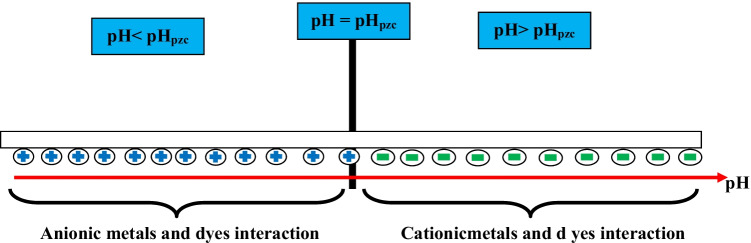
The diagram represents the structural load of MO at different pH values

When pH > pH_pzc_, it indicates the net material surface charge is negative (to promote the adsorption of cationic substances). When pH < pH_pzc_, the net material surface charge reckoned is to be positive (to promote the adsorption of anionic substances). So, this indicates that below this solution pH, the adsorbents are positively charged, while above this solution pH, the surface of the modified adsorbents is negatively charged (see Fig. 8).

The pH_pzc_ of the MO seeds is within the range of 6 to 7 (Araújo et al. 2010b). In another work, the pH_pzc_ of MO seeds was found to be 4.5 by Çelekli et al. (2019). Reddy et al. (2012) found a pH_PZC_ of 3.7 ± 0.2 in the case of modified MO leaves for the removal of Cd(II), Ni(II), and Cu(II). In another work, pH_PZC_ of pristine MO leaves is 5.5 ± 0.2. Prior to pH_PZC_ determination, MO leaves were carefully washed with distilled water to eliminate adhering dirt and particulate materials and dried in the air and then in the oven at 70 °C for 20 h (Imran et al. 2019). These differences in pH_PZC_ values may be due to the procurement of MO from varying origins, the method of analysis of pH_PZC_ as well as the initial treatment, which varies from one researcher to another and other parameters.

### Effect of temperature on sorption by Moringa oleifera

In most cases of studies reviewed in this paper, the biosorption capacity of *Moringa*-based biosorbents is temperature sensitive and is enhanced with increasing temperature. The MO biosorption is affected by temperature moderately at medium temperatures (20–35 °C) and greatly at higher temperatures, and these authors confirmed that the biosorbent (precisely the MO leaves) can be severely denatured at increased temperature (Adeniyi and Ighalo 2019). Although we must take into account that the increasing temperature of adsorbate solution may not necessarily increase sorption active sites over the surface of the adsorbent but may generate a new pathway that enhances the rate of adsorption of the adsorbate onto the adsorbents including biosorbents (Unuabonah et al. 2007).

The process can be endothermic or exothermic. A positive value of ∆H° specifies that the adsorption process is endothermic in nature, while the opposite is realized in the case of sorption of exothermic nature. In the cases of exothermic processes, low temperatures will favor biosorption, while high temperatures will favor biosorption for the endothermic process. In most of the articles reviewed in this work, researchers focusing on studying the effect of temperature concluded that the sorption was endothermic and spontaneous with enhanced randomness (Gautam et al. 2020b). The beneficial mechanism of biosorption at high temperatures is explained by an enhanced diffusion rate at a relatively higher temperature. For the removal of Diuron, the value of the activation enthalpy (Δ*H*°) is <40 kJ/mol, which allows us to suggest that the uptake process follows a physisorption mechanism (de Bezerra et al. 2020). The same behavior was observed in Adebayo et al. (2019). The authors observed the increase in the randomness degree during the adsorption process, which confirmed the physisorption nature of the sorption mechanisms (Adebayo et al. 2019). A decrease in the percentage removal of Cr(IV) from 91.40 to 37.93% using *Moringa stenopetala* seed powder (MSSP) has been observed as temperature increased from 293 to 353 K. Similar observation has been made on adsorption capacities of Cr(VI) ion when the adsorption capacity of MSSP for Cr(VI) was decreased from 1.37 to 0.57 mg/g with increasing temperature from 293 to 353 K. This fall of the adsorbent metal uptake capacity with an increase in temperature might be due to desorption caused by an increase in the available thermal energy. The decrease in adsorption capacity with increasing temperature, therefore, indicates that this biosorption process is exothermic in nature. The negative values of ΔG° and ΔH° confirm the spontaneous and exothermic nature of the sorption of Cr(VI) ion onto both adsorbents. A positive value of ΔS° indicates the increase in the randomness of Cr(VI) ion at the solid-liquid interface of the adsorbents during the sorption process (Badessa et al. 2020). The adsorption capacity increases with increasing temperature from 30 to 50 °C (Gautam et al. 2020b). The thermodynamic study showed that the adsorptive separation of Pb(II) onto synthesized nanosorbent was spontaneous and feasible with enhanced randomness. The values of ΔS° and ΔH° are 84.75 J and 15.66 KJ mol^−1^; a positive value of ΔH° implicated that the adsorption of Pb(II) was endothermic in nature and for the value of the ΔG° is negative showed the spontaneity and practicability of the sorption process. Reduction in ΔG° values with the increase in temperature professed that higher temperature amplified the extent of adsorption of Pb(II). The synthesized nanomaterial based on MO performed efficiently and 94.08% removal of Pb(II) was achieved within 60 min of contact time (Gautam et al. 2020b). For As(V) removal using activated MO, the Δ𝐺° value is negative showing that this biosorption was feasible and spontaneous. While the positive Δ𝐻° values depicted the endothermic nature of the adsorption. The positive Δ𝑆° values revealed the increased randomness at the solid-solution interface (Sumathi and Alagumuthu 2014).

Hence, the biosorption mechanisms can be suggested according to the change in the magnitude of enthalpy of the adsorption (Δ*H*°). During the physisorption process, the values of Δ*H*° are generally in the range of 2.1–20.9 KJ mol^–1^, while the Δ*H*° value of the chemisorption process climbs into a range of 80–200 kJ mol^–1^. Several researchers confirm that the relative strength is electrostatic (6–80 kJ/mol) > hydrogen bonds (4–13 kJ/mol) > van der Waals (2–4 kJ/mol) (Scheufele et al. 2016; Módenes et al. 2017). However, the positive values of entropy of adsorption (∆*S*°) explain the enhanced randomness at the solid-liquid boundary. While the competitive sorption of solvent and adsorbate molecules is randomness sensitive. Logically, adsorbate molecules displace the adsorbed solvent molecules for the capturing of sorption sites exposed to the surface of the adsorbent, which is amenable for the existence of randomness in the system (Gautam et al. 2020b).

### Effect of sorbent dosage on sorption by Moringa oleifera

The sorbent dosage (SD, g/L) is the most critical parameter, which can establish the capability of an adsorbent for any specified sorption of unwanted pollutant and provides an indication about the treatment cost of MO/MO-based biomaterials. The influential parameter “liquid-to-mass ratio, V/m” included in the mass balance equation of the adsorption system for calculating the sorption capacity can be measured in two ways, either at fixed volume (*V*) with a varied mass (*m*_1_,…., *m*_n_) or at a varied volume (*V*_1_, …..,*V*_n_) with a fixed mass (*m*). It was ascertained from the available literature data on MO biosorption that changes in the value of this parameter is strongly subjective to applied concentrations of pollutants, type of pollutant, water chemistry (pH, water quality), and as well as on the conditions used for investigating the effect of this parameter. For example, the investigations on the removal of Cr(VI) using MSSP have shown that the percent removal of Cr(IV) increases from 24.85 to 90.96% with an increase in adsorbent dose from 5 to 20 g/L, while the other factors were kept constant. However, the quantity of adsorbate metal ion per unit weight of the adsorbent decreases with an increase in the adsorbent dose (Badessa et al. 2020).

To identify the mass transfer resistance parameters (e.g., external mass transfer rate, intraparticle surface diffusion, and pore diffusion) controlling the biosorption rate, we must follow the variations in the quantity of solute adsorbed onto an adsorbent as a function of time. Various kinetic models are largely exploited to analyze the determined kinetic data; most of these models are represented in the next part.

## Efficient models for the modeling of biosorption equilibrium and kinetics data

### Modeling of biosorption equilibrium data

The effectiveness of a biosorbent is typically approved by its ability to remove a specific adsorbent irrespective of chemical or physical interactions, batch sorption experiments at constant temperature conditions are carried out. The isotherm of the solid phase adsorbate concentration (*q*_e_) and the liquid phase equilibrium concentration (*C*_e_) is plotted. Various adsorption isotherm models are practiced, and these models provide important information concerning the mechanisms of biosorption and tender information about the retention characteristics of a biosorbent. Additionally, these models allow explaining the distribution of the sorbate between solid and liquid phases at equilibrium conditions (Demey et al. 2018).

The famous theoretical isotherm models that describe the biosorption process are the Freundlich, Langmuir, and Sips models. The Temkin model is most of the time practiced when the temperature effect takes into account*.* The mathematical fit of the experimental data applying these models does not necessarily mean that all the relevant physical assumptions are verified; this can be useful for obtaining additional information on sorption mechanisms. Knowing that the Langmuir model is the most used to explain the homogeneous area, the Freundlich is the best and most reliable to explain the phenomenon of biosorption in heterogeneous surfaces, and the Sips model for heterogeneous and mixed surfaces. The results of Araújo et al. (2010a) confirm the higher heterogeneity of the MO matrix*.* Despite the heterogeneous surface of *Moringa*, the Langmuir isothermic model remains the frequently used model in MO biosorption because of the high adsorbate concentrations used. The Langmuir isotherms (L-shaped) is characterized by a slope, which increases with increasing concentration because the vacant sorption sites decrease as the biosorbent is covered. Such sorption behavior could be explained by the strong affinity of the adsorbent for the adsorbate; in addition, it is due to the availability of active sites at low concentrations, which then decreases with increasing concentration. For example, the nanoadsorbent based on MO has been explored for the effective removal of Pb(II), isotherm analysis revealed that the sorption of Pb(II) onto MO obeyed the Freundlich adsorption isotherm. The maximum adsorption capacities were found to be 49.0, 54.7, and 65.0 mg·g^−1^ at 30, 40, and 50 °C, respectively. The values of separation factor (*R*_L_) were found to be 1.14 × 10^−3^ for 30 °C, 1.34 × 10^−3^ for 40 °C, and 1.73 × 10^−3^ for 50 °C (Gautam et al. 2020b). The removal of As(V) using activated MO at pH 7.0, 140-min equilibrium time, 120 rpm, and 303 K was studied and the *Q*_max_ by using the Langmuir model was 6.23 mg/g. The fitting of the Langmuir isotherm was better relative to the Freundlich isotherm, thus indicating that the applicability of monolayer coverage of arsenic on increasing the temperature increased the arsenic adsorption rate. The equilibrium data was also well described by the Temkin equation further supporting arsenic adsorption on MO as a chemisorption process (Sumathi and Alagumuthu 2014).

Besides, to estimate the suitability degree of each model, we must look at its basic assumptions. For example, the Freundlich and/or Sips model stipulates that pollutant binding to the adsorbent surface is primarily by physical forces (i.e., electrostatic or London–van der Waals forces). The next assumption for the Temkin model is that all sites possess an equal affinity for the adsorbate (Davis et al. 2003). So, assuming that all surface and adsorption sites are equivalent and have the same affinity for a certain solute (surface of biomass is energetically homogeneous*).* The adsorption phenomenon is simply represented by the migration and the occupation of a surface site on a solid (adsorbent) by a pollutant (Davis et al. 2003). Each pollutant must occupy one energy site on the sorbent surface (only bind a single molecule of sorbate), and adsorbed molecules do not interact with each other in a way that influences their sorption behavior. In the biosorption process, at least one of the Langmuir hypotheses is not applicable because always the biomass behavior remains unexplained and anonymous. Generally, in the case of *Moringa*, we have heterogeneous sites, the most suitable model is that of Sips because we have chemical adsorption followed by physical adsorption (multilayer) or Langmuir also, can better represent experimental data.

The Langmuir binding parameter, typically represented by “b,” reflects quantitatively the “affinity” between the sorbent and the sorbate (Holan and Volesky 1994). For the Langmuir isotherm equation, the product of Langmuir maximum capacity parameter and binding parameter (*q*_m_ × *b*) is analogous to a distribution coefficient (L g^−1^) and represents the initial slope of the isotherm curve (Coldebella et al. 2017; Wang et al. 2017).

### Adsorption kinetics

The kinetics of biosorption is an essential knowledge necessary for the practical implementation of a biosorbent because the state of adsorption equilibrium is not accomplished instantly. It is a well-recognized reality, especially for porous biosorbents like MO. The biosorbents with rapid biosorption kinetic properties are desired due to the short contact time between adsorbing ions and biosorbents in the biosorption reactor. The identification of mass transfer resistances influencing the kinetics of biosorbent and optimization of specific biosorbent properties regarding biosorption kinetics can guide us to improve the manufacturing process of biosorbent for rapid kinetics.

The adsorption phenomena of MO can be studied from a kinetic point of view; the experimental data concerning the adsorbed quantity of the pollutant as a function of the contact time variation are important because they provide us additional information on mass transfer resistance. During biosorption, the transfer of adsorbing metal ions takes place from the fluid phase to the active sites of the biosorbents take place in four chronological phases (Worch 2012; Usman et al. 2020b, 2021) as illustrated in Fig. 10. These four chronological phases of adsorption kinetic are

- Transport of metal ion from the solution to the stagnant (boundary) layer enveloping the discrete particle of the biomaterial
- Transfer of metal ions across the boundary layer to the biosorbent’s surface*.* This phase of the sorption kinetics is largely known as film diffusion and represents the displacement of metal ions in the liquid phase in proximity to the solid particle.
- Transfer of metal ions to the active energy sites inside the discrete particle. This phase is overwhelmingly classified as intra-particle surface diffusion or simply surface diffusion in the micropores and macropores of particles.
- Bounding of metal ions to the unoccupied active energy sites available in the discrete particle of a biomaterial, and it is achieved by complexation or precipitation of the metal ion or even by a mixed biosorption mechanism. The adsorbing ion can diffuse from one adsorption site to another either in the free state (after desorption) in the intraparticle liquid phase (migration characterized by a diffusion coefficient *D*_f_) or in the adsorbed state, for an adsorption site to an adjacent site (surface migration). The mechanism of the adsorption process showed that the film diffusion is mostly a rate-determining step (Jabar et al. 2020).

In the available literature, MO adsorption kinetics is explained by traditional kinetics models based on the rate order of chemical reactions. The kinetic models most often used for biosorption experiments are the pseudo-first-order (PFO) model (Lagergren 1898; Barrett et al. 1951; Tien 1994; Srivastava et al. 2006) and the pseudo-second-order (PSO) model (Weber 1963; Do 1998; Ho and McKay 1998). These equations are introduced due to their relatively simple and clear expressions in the application of MO adsorption (Ho and McKay 1999; Katal et al. 2012; Zhang et al. 2013). Equations 10 and 11, respectively, relate to the nonlinear expression of the PFO and PSO models.

10 $${q}_t={q}_{\mathrm{eq}}\left[1-{e}^{-{k}_1t}\right]$$

11 $${q}_t=\frac{q_{\mathrm{eq}}^2{k}_2t}{1+{q}_{\mathrm{eq}}{k}_2t}$$

It should be noted that respective linear forms of Eqs. 10 and 11 are

12 $$\ln \left( qe- qt\right)=\ln\ qe-\frac{k_1}{2.303}\ t$$

13 $$\frac{t}{q_t}=\frac{1}{k_2\ {q}_{\mathrm{eq}}^2\kern0.5em }+\frac{1}{q_{eq}}\ t$$

with *q*_*t*_ and *q*_eq_ are respectively the solid phase quantities of metal ion at a specific time and at equilibrium. 𝑘_1_ and 𝑘_2_ are, respectively, the rate constants of PSO (min^−1^) and PSO (g mg^−1^ min^−1^). It should be noted that rate constants 𝑘_1_ and 𝑘_2_ should be considered apparent rate parameters. A kinetic study by Gautam et al. (2020b) deciphered that equilibrium adsorption for the removal of Pb(II) by MO corresponds more to the pseudo-second-order model than to the first-order model. The first-order rate constant *k*_1_ was found to be −0.0682 min^−1^, whereas the calculated second-order rate constant *k*_2_ was 3.75 × 10^−3^ g mg^−1^ min^−1^ (Gautam et al. 2020b).

For the MO and MO-based biomaterials, nonlinear expressions of the kinetic models have been applied since these biomaterials have heterogeneous surfaces (Benettayeb and Haddou 2021). Indeed, these two models were initially developed for the description of homogeneous reactions; their application to heterogeneous systems means that the intrinsic contribution of diffusion resistance is included in the apparent rate parameters (in the first case of linear modeling, it is considered that the speed is constant during all the adsorption stages, but the apparent speed is a speed made up of several speeds due to the conditions of the adsorption reaction and to the heterogeneous surface of the adsorbent and other conditions directly related to the type of adsorbent). The total adsorption rate is challenged either by the rate of diffusion of the boundary layer or by the rate of intraparticle diffusion or by the rates of the two stages.

## Biosorption mechanisms and key functional groups in the MO surface

**Fig. 9:**
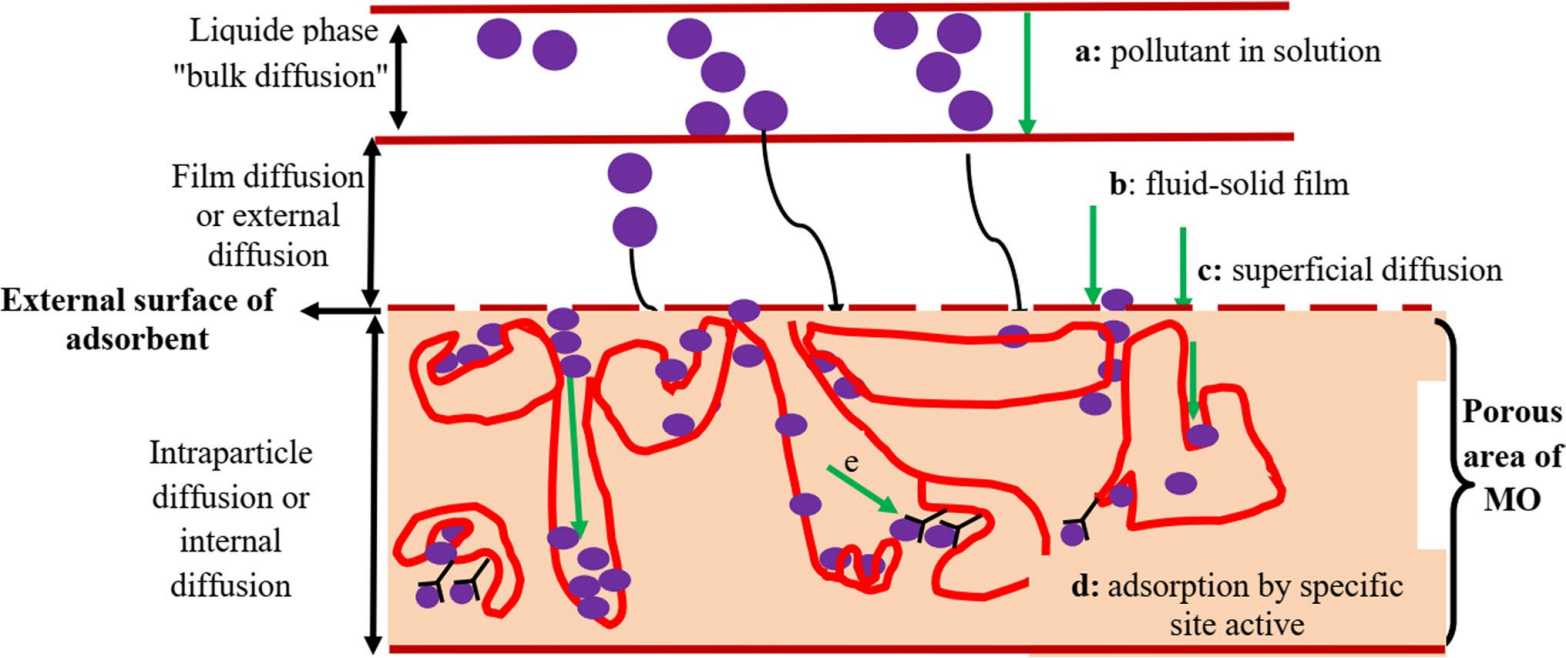
Schematic illustration for the different stages of the transfer of an adsorbate onto a porous area of biosorbent; these steps are inspired by using a work of Weber and Smith (Weber 1972)

The use of *Moringa* as a biosorbent is of environmental and economic interest. However, various conspicuous information gaps remained as an obstacle to understanding the mechanistic pathways responsible for its water purification capabilities using biosorption (Ndibewu et al. 2011; Mnisi and Ndibewu 2017). For this reason, in this review, we have proposed a method of sorption mechanisms of Pb(II), CV, and BG ions on the source of MO and new MO-GEL materials proposed by (Benettayeb and Haddou 2021). Figure 9 gives a schematic illustration for the different stages of the transfer of adsorbate onto a porous area of biosorbent; these steps are inspired by using a work of Weber and Smith (Weber 1972).

For *Moringa*, you can observe that different types of interactions can act simultaneously. Some of the physical and chemical interactions are precipitation, ion exchange, complexation, hydrogen bond, electrostatic interaction, acid-base interactions, and others. In most cases, a combination of these interactions is proposed to explain the biosorption mechanisms of MO like most biosorbents. So, some gaps motivated the research into elucidating the surface and adsorptive properties of the different parts of MO (such as seeds, husks, leaves) to appreciate its sorption mechanisms.

Many functional groups, especially the protein participate in the MO biosorption process. Each of them has a different affinity for adsorbing heavy metal/dyes, and there are several parameters to manage these preferences and affinity. Accordingly, in this part of this review, the biosorption mechanisms and the sites participating in this mechanism for the elimination of different toxic pollutants by different biomaterials have been holistically examined.

According to various work using MO, the FTIR spectrums showed the presence of hydroxyl group (–OH), carboxylic acids (–COOH), protein (–NH_2_), presence of; C=C, esters (–C=O), ethers (>C–O), and the ring involvement or aromatic structure of the compound can be involved in several mechanisms and methods in the biosorption process of metals and dyes like; ion exchange, chelation, physisorption, chemisorption, etc. (Abatal et al. 2021; Benettayeb and Haddou 2021).

For the MO powder, the chemisorption is the main mechanism, which is facilitated and supported by electrostatic attraction and ion-exchange mechanisms, and in some cases, the electrostatic attraction is also the most common mechanism in the biosorption. According to Haghseresht et al. (2009), the ion exchange method is usually used when porous sorbents have the function of cation exchange. Ion exchange occurs between soluble ions such as K^+^, Na^+^, Ca^2+^, NH_4_^+^, and metal ions. Also, according to Hegazy et al. (2021), the hydroxyl (OH) groups, C–H, C=C of alkenes, and C–O of carboxylic acids are the functional groups responsible for metal removal in *Moringa* seed husks.

**Fig. 10:**
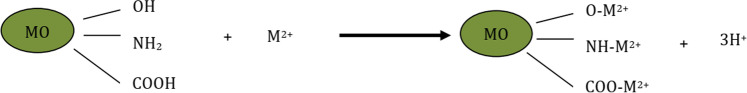
Probable ion-exchange mechanism between MO and metal ions (M^2+^)

**Fig. 11:**
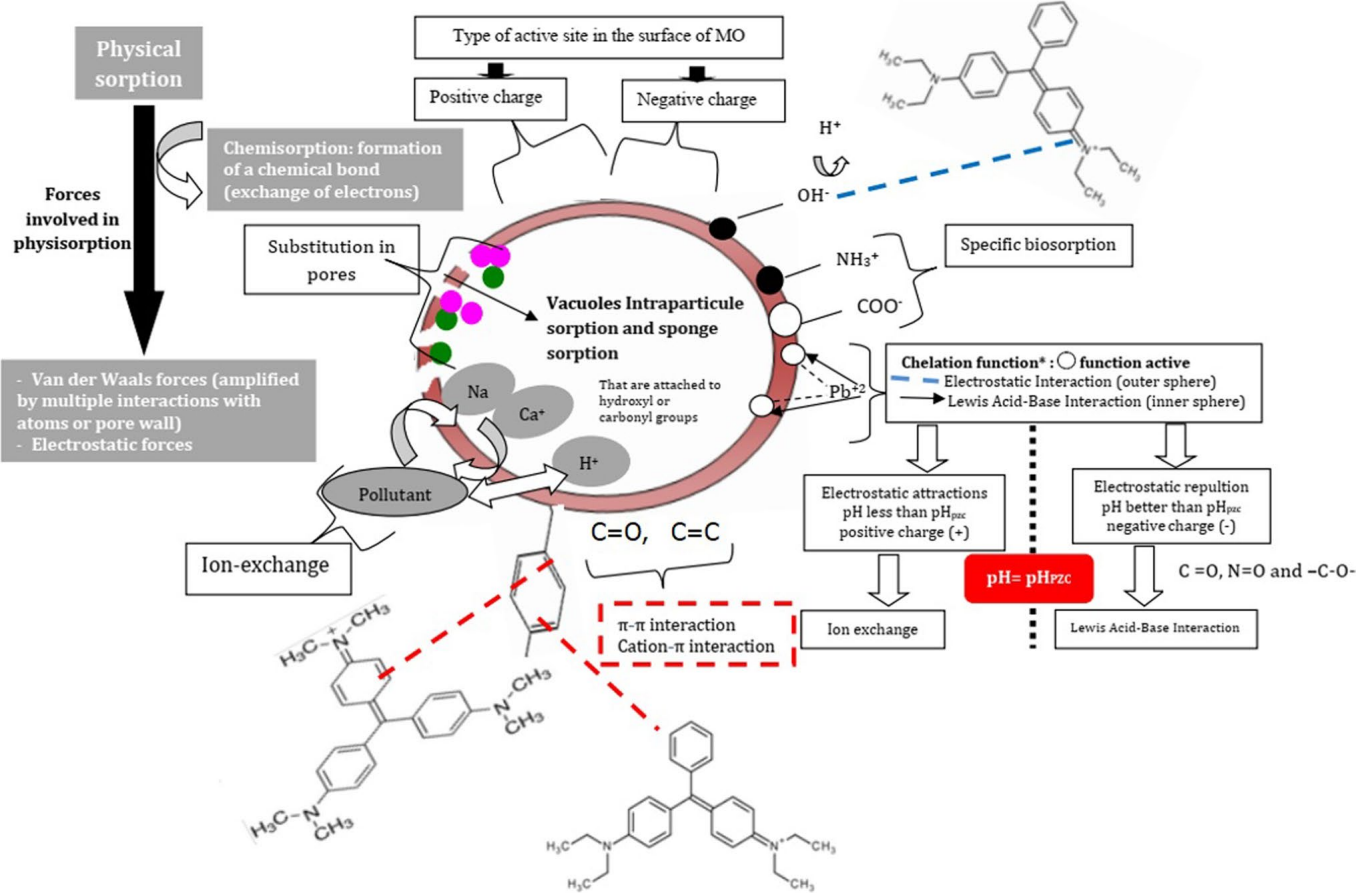
Schematic illustration of the different mechanisms involved in MO biosorption to combat toxic contaminants like CV, VB, and Pb(II) in aqueous solutions

Figure 8 represents the structural load of MO at different pH values. The electrostatic and other possible mechanisms on the surface of MO are illustrated in Figs. 10 and 11. With increasing pH, and when pH < pH_pzc_, the increased H^+^ in solution will react with the surface hydroxyl groups to form some protonated hydroxyl groups. The electrostatic attraction between a protonated hydroxyl group and M^2+^ (for example Pb^2+^) will be favorable for biosorption. The electrostatic characteristics are affected by the existence of surface chemical groups and pH solutions (Moreno-Barbosa et al. 2013). The active sites in surface groups in aqueous media can act for the capture and retention of metal species. Generally, the biosorption of Pb(II) can be influenced by the interaction mode with the solid surface (de Oliveira et al. 2019), ion-exchange mechanism, and the electrostatic characteristics between the biosorbent and the adsorbate. For example, the capacity of MO to remove heavy metals has highlighted amino acid-metal interactions responsible for the adsorption phenomenon (Subramanium et al. 2011; Nand 2012). The presence of more –OH groups on the sorbents’ surface creates more possibility of Pb–OH ion exchange (Fig. 11), and nitrogen and oxygen functional groups can provide many active adsorption sites to form strong chelation with lead ions in the aqueous solution.

Based on available literature on MO as biosorbent, Fig. 11 shows the possible mechanisms involved in the biosorption of cationic dyes (for example, CV, VB) and Pb(II). Generally, the positively charged pollutant in water is first attracted to the MO/MO-based biomaterials surface by the electrostatic force. Then, the metal ions or dyes diffuse in the entry of the pores and after inside in the pores by ionic diffusion, then they are put in contact with the various functional groups. Finally, the ions of metals or dyes are fixed with one of these groups (according to their preferences, their nature, and class) containing nitrogen –N, oxygen –O, or sulfur –S by chelation and/or ion exchange, forming a stable binding structure (metal ions according to their types and preferences, size, etc.), which is one of the possible paths of pollutants adsorbed by MO and MO-based biomaterials. View that the complexity of the mechanisms, which remains difficult to understand because it is mixed, and we find several types of adsorption and reaction at the same time (Swelam et al. 2018; Tang et al. 2018).

It is relevant to highlight that the MOS contains polyelectrolyte groups that can also contribute to the adsorption of positive metal ions and can form a bridge between the anionic polyelectrolyte and negatively charged protein functional groups on the colloidal particle surface (Steinnes 1995; Abdeen 2018).

## Conclusion and prospects of MO as biosorbent

As a part of the resolution of the environmental problems related to the presence of organic (dyes) and inorganic (heavy metal ions) pollutants in wastewater, this review concludes that the seeds, husk, leaves, and bark of the MO plant can efficiently be applied for the removal of aforementioned pollutants in water, and it has substantial potential that serves as an alternative sorbent to chemically synthesized materials in water purification. This review further explores the possibility of the improvement of the MO for use in industrial wastewater treatment. Therefore, in this review, we tried to answer the question “why there is a growing interest to use MO in the field of biosorption?”, “what are the important properties of this plant?” and clarify the vision of readers on the miraculous properties of the MO.

Thanks to several pieces of research, all revised results we were able to draw the following conclusion and all of these researchers confirming that

1. MO is a plant, and its growing plantation can participate in rural economic development.
2. The interest in the use of MO is due to several advantages such as it is a green biosorbent available in rural areas, an environment-friendly alternative biosorbent for the remediation of some contaminated waters. Recent research works have confirmed that MO has several advantages over commonly used biosorbents. For example, powdered MOS contain cationic polyelectrolytes, which act as a natural flocculent to clarify even the most turbid water and achieve biosorption, low operating cost, production of biodegradable sludge, and lower sludge volume and pH of the water unchanged.
3. The different characterization methods used by the researchers revealed the presence of different functional groups such as hydroxyl, amine, and carbonyl indicating the complex nature and justifying the biosorption capacity of MO. These functional groups have enabled the adsorption process because they offer active sites responsible for biosorption.
4. The different physicochemical mechanisms responsible for the retention of heavy metals by MO are presented in this review and thanks to the heterogeneous rich surface, the mechanism of biosorption by MO is rich and includes electrostatic attraction and ion exchange.
5. In order to increase the pH range and to check the precipitation phenomena, it is necessary to first carry out a pH study and a verification of the pH_PZC_ and necessary to give information about the surface charge at different pH.
6. Literature work on biosorption thermodynamics indicated that most of the sorption process was endothermic, and the negative values of ΔG° showed a spontaneous and favorable biosorption process.
7. Some theoretical models are exposed in this work; the validation of the modeling of the experimental results by one or the other model does not necessarily mean that the hypotheses linked to these models are validated; the results and the interpretations also depend on the experimental conditions. In MO biosorption, generally, the chemisorption is followed by physisorption, the latter making the surface fragile with additional layers of pollutants. This type of adsorption allowed the ionic species to adhere to active sites of adsorbent and other types of physicochemical interaction. Unfortunately, despite the richness of this plant, it is humiliating by most research, and a bit of good research has been developed for biosorption using this plant. However, further studies are needed on the use of MO to remove toxic dyes and heavy metals, and researchers are in quest of novel environment-friendly techniques for the modification of MO in order to not affect its basic efficiency and to improve these properties.

In this work, we encourage researchers to find new ways of modifying the structure of different parts of MO, especially the leaves and seeds in order to improve these basic properties and increase their efficiency against toxic pollutants. We suggest the utilization and enhancement of new physical forms enhance the use of this free natural source.

More in-depth research will be desirable in the future for a promising valorization of these biosorbents, which could compete with commercial biosorbents in the treatment of wastewater, in particular, the effluents of textile industries to preserve a healthy and livable environment. In addition, a dynamic study would be necessary before a possible transition to real conditions.

In future studies, adsorption kinetics models such surface diffusion model and pore diffusion model should be employed to elucidate the mass transfer processes controlling the biosorption process. This would also us to identify the *Moringa*-derived biosorbents with rapid adsorption kinetics. Similarly, based on the quantification results on MO biosorption kinetics, different application-orientated water treatment strategies relying on adsorption such as fixed-bed adsorption columns and adsorption-membrane hybrid systems can be explored.

1. *Moringa* species other than MO such as *Moringa stenopetala* and *Moringa drouhardii* should be comprehensively explored for use in wastewater treatment for heavy metal ions.
2. We encourage researchers to synthesize other generations of MO either by cold gelation method (using olive oils) or hot (ionic gelation) to form beads and test them in comparative studies in batches and in dynamic mode for a future application in the treatment of wastewater (in order to optimize the parameters of operation) to pass following to real applications in the chemical industry.

## Data Availability

All data are mentioned in the body of the manuscript, tables, and figures.
